# Quantification of bacteria by in vivo bioluminescence imaging in comparison with standard spread plate method and reverse transcription quantitative PCR (RT-qPCR)

**DOI:** 10.1007/s00203-021-02458-5

**Published:** 2021-06-29

**Authors:** Katarína Briestenská, Miriam Mikušová, Karolína Tomčíková, František Kostolanský, Eva Varečková

**Affiliations:** Biomedical Research Center of the Slovak Academy of Sciences, Institute of Virology, Dúbravská cesta 9, 845 05 Bratislava, Slovakia

**Keywords:** *Streptococcus pneumoniae*, Mouse model, Spread plate method, RT-qPCR, In vivo bioluminescence imaging, Correlation

## Abstract

In vivo bioluminescence imaging (BLI) offers a unique opportunity to analyze ongoing bacterial infections qualitatively and quantitatively in intact animals over time, leading to a reduction in the number of animals needed for a study. Since accurate determination of the bacterial burden plays an essential role in microbiological research, the present study aimed to evaluate the ability to quantify bacteria by non-invasive BLI technique in comparison to standard spread plate method and reverse transcription quantitative PCR (RT-qPCR). For this purpose, BALB/c mice were intranasally infected with 1 × 10^5^ CFU of bioluminescent *Streptococcus pneumoniae* A66.1. At day 1 post-infection, the presence of *S. pneumoniae* in lungs was demonstrated by spread plate method and RT-qPCR, but not by in vivo BLI. However, on the second day p.i., the bioluminescent signal was already detectable, and the photon flux values positively correlated with CFU counts and RT-qPCR data within days 2–6. Though in vivo BLI is valuable research tool allowing the continuous monitoring and quantification of pneumococcal infection in living mice, it should be kept in mind that early in the infection, depending on the infective dose, the bioluminescent signal may be below the detection limit.

## Introduction

*Streptococcus pneumoniae*, also known as pneumococcus, is a leading cause of community-acquired pneumonia and accounts for significant morbidity and mortality worldwide (Brooks and Mias 2018; Torres et al. 2018; Feldman and Anderson 2020). As an opportunistic pathogen, *S. pneumoniae* colonizes mucosal surfaces of the upper respiratory tract (URT) in humans. Although this colonization is usually asymptomatic in healthy individuals, it marks the first step in the development of invasive pneumococcal disease. The transition from asymptomatic colonization to disease is highly associated with preceding or concomitant respiratory viral infections, especially influenza, and pneumococcal ability to evade the early components of the host immune response. Changes in the URT microenvironment and inflammation trigger *S. pneumoniae* dissemination to the middle ear cavity (causing otitis media), the lower respiratory tract (causing pneumonia), the bloodstream (causing sepsis) and/or the meninges (causing meningitis) (Kadioglu et al. 2008; Short et al. 2012; Marks et al. 2013; McCullers 2014; Chao et al. 2015; Weiser et al. 2018).

A mouse model of pneumococcal pneumonia (Chiavolini et al. 2008; Borsa et al. 2019) was utilized to clarify mechanisms of pneumococcal pathogenesis and to assess the efficacy of novel antibiotics and vaccines, as well as to study various aspects of pathogenesis of dual infection with influenza virus and *S. pneumoniae* (McCullers and Webster 2001; McCullers and Rehg 2002). However, it is essential not only to use a suitable animal model, but also to select the appropriate assessment method (or combination of methods). Therefore, the aim of the present study was to evaluate the pneumococcal infection in mice using different methods: the standard culture-based method (spread plate method), the molecular biology technique (reverse transcription quantitative PCR; RT-qPCR) and the non-invasive imaging method (in vivo bioluminescence imaging). The ability to quantify bacteria by these methods was compared.

## Materials and methods

### Bacterial strain and growth conditions

*S. pneumoniae* A66.1 (serotype 3) bearing the lux transposon cassette, Tn4001 luxABCDE(Km^r^), was provided by Dr. Jonathan A. McCullers from St. Jude Children’s Research Hospital (Memphis, TN, USA). *S. pneumoniae* bacteria were grown to mid-log phase (OD_600_ = 0.6) in Todd-Hewitt broth (Sigma Aldrich) containing 400 μg/mL kanamycin (AppliChem) at 37 °C and 5% CO_2_ without shaking. Titer (CFU/mL) of *S. pneumoniae* was determined by spread plate method on nutrient agar no. 2 (Sigma Aldrich) supplemented with 10% defibrinated sheep blood and 400 μg/mL kanamycin (AppliChem).

### Mice and infection

Six- to eight-week-old female BALB/c mice (*n* = 15) purchased from the Faculty of Medicine of the Masaryk University (Brno, Czech Republic) were used in this study. Animals were treated according to the standards of the European Union and State Veterinary and Food Administration of the Slovak Republic (SVFA SR). Fundamental ethical principles including animal welfare requirements were respected. All experimental procedures were approved by the SVFA SR before the study was begun (permission number 3932/17-221). Mice were anesthetized with isoflurane using the XGI-8 Gas Anesthesia System (PerkinElmer) and inoculated intranasally with 40 μl of phosphate-buffered saline (PBS) containing 1 × 10^5^ CFU of bioluminescent *S. pneumoniae*. Mice were weighed and monitored daily for signs of disease.

### In vivo bioluminescence imaging (BLI)

On days 1, 2, 3, 6 and 9 post-infection (dpi), mice (*n* = 3 per interval) were anesthetized with isoflurane using the XGI-8 Gas Anesthesia System (PerkinElmer) and imaged in dorsal and ventral positions using the IVIS^®^ SpectrumCT In Vivo Imaging System (PerkinElmer). For image acquisition and analysis of bioluminescent signal, Living Image software (version 4.5.5; PerkinElmer) was used. As recommended in Living Image software user’s manual, minimum value for counts was set to 600 to ensure signal that is well above the noise.

### Determination of bacterial titers in lungs

Immediately after in vivo BLI, mice were euthanized by cervical dislocation under deep tiletamine/zolazepam anesthesia and lungs were aseptically harvested and washed two times in PBS. Lungs were weighed and promptly homogenized in cold PBS using T 10 basic ULTRA-TURRAX^®^ homogenizer (IKA) to achieve 20% homogenates. Lung homogenates were pelleted at 1000×*g* for 5 min at 4 °C. To determine the bacterial titers in lungs (CFU/mg of tissue), the samples were serially diluted tenfold and plated on nutrient agar no. 2 (Sigma Aldrich) supplemented with 10% defibrinated sheep blood and 400 μg/mL kanamycin (AppliChem). Plates were incubated at 37 °C in a 5% CO_2_ humidified atmosphere for 24 h. Bioluminescence of grown colonies was verified using the IVIS system.

### Two-step RT-qPCR

Total RNA was isolated from 200 µl of lung homogenate supernatants using TRI Reagent™ Solution (Invitrogen) according to the standard protocol. RNA pellets were resuspended in 30 μl of DEPC-treated water (Ambion). The quantity and quality of RNA samples were assessed by NanoDrop 2000c Spectrophotometer (Thermo Scientific). Subsequently, 2 μg of total RNA was reverse transcribed in a 20 μl reaction volume using the RevertAid First Strand cDNA Synthesis Kit (Thermo Scientific) following the manufacturer’s instructions. The resulting cDNA was diluted fivefold in nuclease-free water. Then, mouse β-actin was amplified as an internal control. Thus, verified cDNA was used as a template in qPCR with primers specific for the α subunit of luciferase gene (*luxA*) (Fw: 5′-GCATATTTACTTGGCGCGACT-3′; Rev: 5′-TGCGCCACCTCTGCTATAC-3′). qPCR amplification was performed on a StepOnePlus™ Real-Time PCR System (Applied Biosystems) using Maxima SYBR Green/ROX qPCR Master Mix (Thermo Scientific) according to the manufacturer’s instructions. qPCR reactions contained 3 µl of cDNA as a template. qPCR standard curve was generated using tenfold serial dilutions of the standard—10^7^–10^1^ copies were used as templates in qPCR mixtures. The amplification conditions were as follows: 10 min at 95 °C, 40 cycles of 15 s at 95 °C and 1 min at 60 °C. The results were analyzed using StepOne software (version 2.3; Applied Biosystems).

### Statistical analysis

Correlation between methods was calculated as Pearson’s correlation coefficient *r* along with R square and *p* value (two-tailed). The statistics and graphs were made using the software Prism 7 (GraphPad Software Inc.).

## Results

**Fig. 1:**
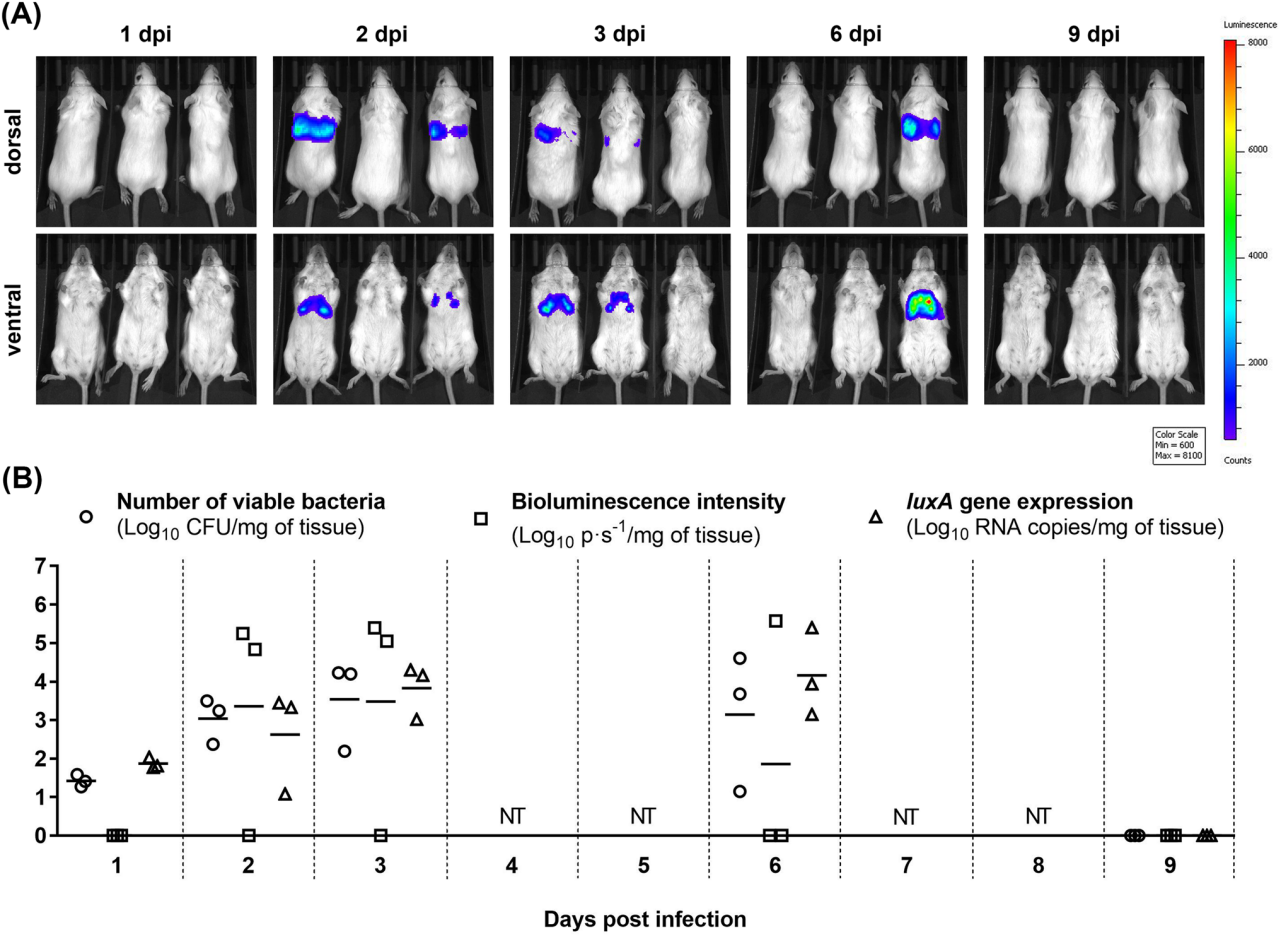
Evaluation of pneumococcal infection in mice using different methods. BALB/c mice were intranasally infected with 1 × 10^5^ CFU of bioluminescent *S. pneumoniae* A66.1. At indicated days post-infection (1, 2, 3, 6 and 9 dpi), **A** mice (*n* = 3) were imaged by the IVIS^®^ SpectrumCT In Vivo Imaging System (PerkinElmer) and **B** photon flux was quantified from bioluminescent images. Immediately after in vivo imaging, mice were euthanized and lungs were collected. Bacterial titers in lungs were determined by inoculation of samples onto the sheep blood agar plates. Expression of *luxA* gene in the lungs was analyzed by RT-qPCR. Each symbol represents a value from an individual mouse and the bars indicate average values. *NT* not tested

The aim of this study was to compare the ability to quantify bacteria by three different techniques during the course of infection with bioluminescent *S. pneumoniae* in BALB/c mice. At 1 dpi, mice intranasally infected with 1 × 10^5^ CFU of *S. pneumoniae* did not show any signs of infection and no signal was detected in mice by in vivo BLI (Fig. 1A). However, viable bacteria were recovered from lung tissue samples even at 1 dpi (Fig. 1B). These samples were also found to be positive by two-step RT-qPCR targeting *luxA* gene (Fig. 1B). At 2 dpi, mild symptoms of disease, such as ruffled fur and shivering, were observed in mice. Bioluminescent images were characterized by strong signals from the thorax but no other anatomical locations in two of three mice (Fig. 1A). Photon flux (photons per sec) was then quantified from selected and defined areas within the ventral images of each mouse (as described in Francis et al. 2001) and the results are presented in Fig. 1B. These observations suggested that only two mice had an established pneumococcal lung infection even though all three mice were infected with the same dose of *S. pneumoniae*. In the lungs of mouse with no evident luminescent signal, the presence of bioluminescent *S. pneumoniae* was proved by spread plate method and RT-qPCR, but bacterial burden and expression level of *luxA* (Fig. 1B) was lower compared to other two mice. The interindividual variability among mice indicated differences among mice to cope with infection. At 3 dpi, the bacterial titers in lungs reached peak (mean = 3.54 log_10_ CFU/mg of tissue, SD = 1.17 log_10_ CFU/mg of tissue) (Fig. 1B) and mice developed severe clinical symptoms, including hunched posture, decreased activity, and labored breathing. Moreover, macroscopic examination of lungs revealed some areas of hemorrhage. These findings were consistent with BLI results as the strong signals were seen in the lungs of two mice (Fig. 1A, B). Similar to the previous time point (2 dpi), the mouse that showed no bioluminescent signal at 3 dpi was the one with the lowest bacterial burden in the lungs. At 6 dpi, one mouse had no obvious signs of disease and only minimal pathological changes macroscopically visible in the lungs. The other two mice exhibited serious disease symptoms. These two sick mice had high bacterial titers and high levels of *luxA* expression (Fig. 1B) in the lungs. Surprisingly, bioluminescent image (Fig. 1A) displayed severe pneumonia only in one mouse. On gross examination, the lungs of mouse with robust bioluminescent signal were highly edematous and hemorrhagic, while the lungs of the other mouse were heavy, gray discolored and filled with mucus. Therefore, we hypothesize that mucus accumulated in the lungs could cause the inhibition of the bioluminescent signal. At 9 dpi, all three mice appeared healthy with normal activity, behavior, and texture of the fur. Lung samples from these mice were tested negative for *S. pneumoniae* by all methods used (Fig. 1B).

The data on *S. pneumoniae* in lung samples obtained by different methods were plotted against each other and the Pearson’s correlation coefficient (*r*) was calculated. Despite some differences in positivity of infection determined by spread plate method and in vivo BLI and variability of data obtained from individual mice at each time point, the overall correlation between these methods was strongly positive (*r* = 0.90; *p* ≤ 0.0001). Results of the Pearson’s correlation test for other method pairs (spread plate method vs. RT-qPCR, in vivo BLI vs. RT-qPCR) are presented in Table 1.

**Table 1:** Correlation between data on *S. pneumoniae* in mouse lungs determined by different methods—spread plate method (viable bacterial count), in vivo bioluminescence imaging (bioluminescent signal) and RT-qPCR (*luxA* expression)

	Pearson’s correlation coefficient *r*	*r*^2^	Significance, *p*
Viable bacterial count vs. bioluminescent signal	0.90	0.81	≤ 0.0001
Viable bacterial count vs. *luxA* expression	0.91	0.82	≤ 0.0001
*luxA* expression vs. bioluminescent signal	0.78	0.61	≤ 0.01

## Discussion

Culture-based and molecular biology techniques are widely used for the quantification of bacteria. However, both techniques suffer from a number of disadvantages. One of them is day-to-day variability in bacterial counts associated with random selection and killing of mice (Ogunniyi et al. 2018). This could be eliminated by the imaging of bioluminescent reporter bacteria in living mice using a cooled charge-coupled device (CCD) camera. The bioluminescence intensity is proportional to the microbial concentration; therefore, in vivo BLI can be used not only for qualitative but also for quantitative analysis. This technique allows for non-invasive real-time monitoring of infection and tracking of disease progression in an individual animal over time without the need of serial killing of animals, thus the number of research animals is reduced. Moreover, sites of infection not easily sampled by dissection (such as the middle ear) can be visualized and previously overlooked sites can be potentially revealed (Short et al. 2011; Warawa and Lawrenz 2014; Avci et al. 2018).

Our study aimed to determine the bacterial burden in lungs of mice infected with bioluminescent pneumococci using conventional spread plate method, RT-qPCR and in vivo BLI, and to compare quantification capabilities of these methods. Moreover, the Pearson’s correlation test was performed for each assay pair (Table 1). Francis et al. (2001) also evaluated bioluminescent *S. pneumoniae* A66.1 infection in a mouse pneumococcal lung model using in vivo imaging technique. They detected a strong bioluminescent signal from the thorax of mice as early as 20 h post-infection. However, the design of study by Francis et al*.* was different from ours—they used higher inoculum dose (approximately 1 × 10^6^ PFU in 20 µl per mouse), they introduced pneumococci into the lungs of BALB/c mice by intratracheal inoculation using a ball-tipped gavage needle, and they monitored bioluminescent infection only for 48 h (Francis et al. 2001). We used tenfold lower dose of inoculum to induce morbidity but not mortality in mice so that we could monitor the course of infection over longer period of time (9 dpi). In addition, pneumococci were administered into the nostrils of mice in 40 µl of PBS. In our study, the presence of *S. pneumoniae* in lungs of mice at 1 dpi was demonstrated by spread plate method and RT-qPCR targeting *luxA* gene. In vivo BLI, however, failed to detect early pneumococcal infection (1 dpi), which could be due to the lower inoculum dose and the “dilution effect” of intranasally administered bacteria within the respiratory tract, making them undetectable by BLI (Henken et al. 2010). Furthermore, various biophysical parameters and factors have been described to affect the sensitivity of BLI, including mouse fur, tissue pigmentation, vascularization and depth from the surface (Badr 2014; Troy et al. 2004; Zinn et al. 2008). If necessary, the sensitivity of BLI method can be increased by imaging excised target organs ex vivo (Henken et al. 2010; Gabrielli et al. 2015). However, ex vivo imaging is invasive and does not allow repetitive assessments of an individual animal (Inoue et al. 2006).

At later time points (2–6 dpi) in our experiment, when the bacteria grew to such an extent that the bioluminescent signal was detectable, the photon flux values strongly correlated with bacterial titers (determined by spread plate method) and the number of RNA copies (determined by RT-qPCR) in lungs of mice.

Taken together, in vivo BLI represents a valuable research tool for qualitative and quantitative monitoring of long-term pneumococcal infections non-invasively in living mice. However, it should be kept in mind that early in the infection, the bioluminescent signal may be below the detection limit. In such cases, additional techniques such as RT-qPCR should be considered to examine the presence of bacteria in tissues.
